# Perceived general health in relation to oral health status in a rural Kenyan elderly population

**DOI:** 10.1186/s12903-021-01525-z

**Published:** 2021-03-24

**Authors:** Hideki Fukuda, Yoshihiko Hayashi, Kazuo Toda, Satoshi Kaneko, Evelyn Wagaiyu

**Affiliations:** 1 National Institute of Public Health, 2-3-6 Minami, Wako City, Saitama Prefecture 351-0197 Japan; 2 Graduated School of Biomedical Sciences, Nagasaki University, 1-7-1 Sakamoto, Nagasaki City, Nagasaki Prefecture 852-8588 Japan; 3 Department of Ecoepidemiology, Institute of Tropical Medicine, Nagasaki University, 1-12-4 Sakamoto, Nagasaki City, Nagasaki Prefecture 852-8523 Japan; 4 School of Dental Sciences, University of Nairobi, P.O. Box 30197-00100, Nairobi, Kenya

**Keywords:** Perceived general health, Satisfactory mastication, Tooth loss, Periodontitis

## Abstract

**Background:**

This study aimed to determine the present oral health status of the rural Kenyan elderly population and to investigate whether oral health status is associated with the perceived general health.

**Methods:**

A total of 131 individuals aged 65 years and over in Mbita Constituency, Homa Bay County in Kenya were randomly selected and visited at home. The home visit study, which was conducted from 2014 to 2016, included oral examination by a dentist and administration of a self-reporting questionnaire. The number of teeth present and functional tooth unit (FTU) points were calculated using the dental chart. Perceived general health, subjective masticatory ability, and self-reported periodontal symptoms were collected using a questionnaire sheet. Fisher’s exact test and nonparametric test were used to determine the difference in percentage and means. The odds ratio of good general health based on the participants’ masticatory satisfaction was calculated by logistic analysis.

**Results:**

Satisfactory mastication was dependent on the number of teeth present, FTU points, and self-reported “gum bleeding” and “tooth mobility”. Furthermore, satisfactory mastication was associated with perceived general health status independent of sex and age. The adjusted odds ratio of good perceived general health was 2.29 (95% confidence interval 1.05–4.99) for participants who had the subjective masticatory ability.

**Conclusion:**

Among the Kenyan elderly population, satisfactory mastication was related to the number of teeth present, FTU points, and self-reported periodontal symptoms. Furthermore, satisfactory mastication was associated with perceived general health status independently.

## Introduction

Good oral health status in the elderly should be one of the priority health issues in the aging society because of its impact on general health. Impaired dentitions due to extensive tooth loss without prosthodontic care have been shown to affect the dietary selection and also lead to difficulty in chewing hard foods [1–7]. These conditions could lead to an increase in the risk of low protein [8, 9], low energy intake [10, 11], and frailty [12]. According to the prospective studies for the elderly, keeping a greater number of teeth and wearing dentures showed lower mortality rate [13–15]. In Kenya, the percentage of 65 years and over is only 2.4% in 2019. It is gradually increasing and it will reach 3.4% in 2030 [16]. The elderly rate will be still smaller than other industrial countries, however, it will be imperative for the Kenyan health system to take measures to address dental health issues for the coming elderly society.

To promote a healthy lifestyle in the elderly, their oral health status should be in good condition so that chewing is not a problem. Inadequacy in mastication, specifically in elderly individuals, naturally lead a poorly oral health related quality of life (QOL). The oral health related QOL is associated with their perceive general health in the elderly [17, 18]. The perceived general health is known as a simple and good indicator to measure the risk of mortality in the elderly. DeSalvo et al. reported the persons with “poor” perceive general health had higher mortality risk compared with persons with “excellent” [19].

Manji et al. [20] and Sanya et al. [21] reported dental caries and periodontal diseases were common causes of tooth lost among Kenyans. Pengpid et al. [22] revealed that 13.7% of participants reported poor self-rated oral health with nationwide cross-sectional study. However, the previous papers were conducted about 20 years ago and the later did not deal with perceived general health. There is hardly any published information on the association between oral health status and perceived general health status in the Kenyan elderly with a systematic field research design. Hence, this study aimed to determine the present dental health status in a rural Kenyan elderly population with limited dental services and to investigate whether dental health status is associated with the perceived general health among them.

## Methods

### Design and participants

The Institute of Tropical Medicine at Nagasaki University established the Health and Demographic Surveillance System (HDSS) in 2006 in Mbita Constituency, Homa Bay County in Kenya, which is located approximately 300 km west of Nairobi, Kenya. Mbita Constituency belongs to Homa Bay County. The center of Mbita is about 40 km away from the center of Homa Bay. Kenya Population and Housing Census 2019 [23] showed that most households are employed in farming in Homa Bay County. The percentage of households in farming is 74%, which is larger than the national average of 52% and 2% in Nairobi. It is also reported that the percentage of households using solar energy is 53%, which is larger than the national average of 19% and 0.2% in Nairobi. The HDSS program recruited 11,182 households and 55,929 inhabitants on July 1, 2011 [24]. The HDSS collects periodically vital events such as deaths and births of residents, living environment such as water supply and toilets, and property such as cars and televisions by field interviews. From the latest list of 434 recruited individuals aged 65 and over in the HDSS, 150 elderly persons were randomly selected with computer program from four sub-locations in Mbita Constituency by the staff in charge of HDSS. The sampling rate of this study was 35% (150/434). The home visit study was conducted from 2014 to 2016 in the following sub-locations: Mbita center and south sub-location in 2014, east sub-location in 2015, and middle-south sub-location in 2016. Before commencing the home visit study, community health workers engaged in the HDSS program visited the participants’ houses, explained the purpose of the research, and made an appointment for the home visit. Informed consent and questionnaire sheets were obtained at the time of the home visit by the authors after the purpose of the study was comprehensively explained to the participants. We asked the participants to fill out all questionnaire items. If the participants could not read or understand the contents of questionnaire items, a community health worker who attended with the research team interviewed him/her about the questionnaire items.

A total of 131 participants provided informed consent, received a dental examination, and completed a questionnaire (collecting rate is 87.3%). Remaining 29 subjects transferred to other places and/or died. One of the authors, a dentist (H.F.), examined the dental status of the participants by assessing the participants’ teeth using a disposable dental mirror and a portable light. The examination was performed outside the homes with the participants seated on an ordinary chair under direct sunlight. Dental status was assessed using the World Health Organization standards. The number of teeth present was counted, including the number of sound teeth, decayed teeth, and roots of the teeth. The present teeth ranged from 0 to 32. Functional tooth unit (FTU) are points derived from adding the number of pairs of molar and premolar [25]. One pair of molars and one pair of premolars are equivalent to 2 points and 1 point, respectively, based on FTU. FTU range from 0 to 12 points per participant. If retained dental roots of the molars and/or premolars were observed, they were not counted as a pair.

Data regarding perceived general health, masticatory ability, and self-reported periodontal symptoms were collected with a questionnaire sheet. Perceived general health was measured by answering “good,” “average,” and “bad” to the question “How is your general health?” Perceived general health was divided into the following two categories: “good/average” and “poor.” Data on subjective masticatory ability was collected from the self-recorded questionnaire: “Can you masticate (bite) sufficiently, now?”. Self-reported periodontal symptoms “gum bleeding” and “tooth mobility” were also collected using self-recorded questionnaires: “Have you ever had the following problems in your mouth?”. If any participant had severe tooth pain and oral lesions, the Kenyan dentist (E.W.) prepared to prescribe medication and refer the participant to the nearest dental facilities. However, none of the participants experienced severe tooth pain requiring medication or referral. A visual oral examination was performed without periodontal probing. Therefore, measuring the periodontal pocket depth was not possible. Traditional extraction of lower anterior incisor and canine teeth (33–43), which is common in the elderly population, was verbally confirmed by the participants.

### Data analysis

Percentage of participants with self-reported periodontal symptoms and the mean number of teeth and FTU points by subjective masticatory ability were compared. A difference in percentages was tested using the chi-squared test and Fisher’s exact test. The mean number of teeth present was not a normal distribution. Therefore, the difference in the number of teeth present and FTU points based on the participants’ characteristics and subjective masticatory ability was verified using the Mann–Whitney U test and the Kruskal–Wallis test. In order to examine the relationship between perceived general health and their masticatory satisfaction, the odds ratio of good general health based on the participants’ masticatory satisfaction was calculated by logistic analysis. All statistical analyses were performed using the IBM SPSS version 20.0. The level of significance was set at 5%.

### Ethics approval

This study was conducted in full accordance with the World Medical Association Declaration of Helsinki. The study was approved by the ethics and research committee of the Kenyatta National Hospital/University of Nairobi (P328) on August 7, 2013.

## Results

The mean age of the participants was 75.0 years in men and 75.6 years in women. There was no significant difference in age and sex among the participants (Table 1).

**Table 1:** Mean age and percentage of age class by sex

	Age		*P* value^b^	Age class										*P* value^c^
	Mean	(SD^a^)		65–69 years		70–74 years		75–79 years		80 years and over		Total		
				N	%	N	%	N	%	N	%	N	%	
Male	75.0	(7.6)	0.65	13	28.3	10	21.7	11	23.9	12	26.1	46	100.0	0.81
Female	75.6	(6.1)		19	22.4	17	20.0	26	30.6	23	27.1	85	100.0	
Total	75.4	(6.7)		32	24.4	27	20.6	37	28.2	35	26.7	131	100.0	

^a^Standard deviation

^b^T-test

^c^Chi-square test

A total of 86.3% of participants had the lower anterior incisor and canine teeth (33–43) extracted traditionally. The percentage of traditional extraction increased significantly with increasing age (59.4% and 94.3% in elderly participants aged 65–69 years and 80 years and over, respectively) (Table 2). The percentage of self-reported “tooth mobility” and “gum bleeding” was insignificantly different in terms of sex and age. The number of present teeth and FTU points significantly decreased with increasing age.

**Table 2:** Percentage of traditional examination and self-reported periodontal symptoms and mean number of tooth and FTU points by sex and age

	Number	Traditional extraction		*P* value^a^	Tooth mobility		*P* value^a^	Gum bleeding		*P* value^a^	Present teeth		*P* value^b^	FTU^c^		*P* value^b^
		N	%		N	%		N	%		Mean	Median		Mean	Median	
*Sex*																
Male	46	34	73.9	< 0.01	26	56.5	0.46	16	34.8	0.15	23.4	25	0.06	7.5	9	0.81
Female	85	79	92.9		54	63.5		41	48.2		21.2	24		7.4	8	
*Age class*																
65–69	32	19	59.4	< 0.01	18	56.3	0.87	17	53.1	0.31	26.2	26	< 0.01	9.5	10	< 0.01
70–74	27	24	88.9		17	63.0		14	51.9		23.0	24		7.7	8	
75–79	37	37	100.0		22	59.5		13	35.1		20.8	23		7.1	8	
80–	35	33	94.3		23	65.7		13	37.1		18.6	23		5.7	6	

^a^Fisher's exact test/chi-square test

^b^Mann–Whitney U test/Kruskal–Wallis test

^c^Functional tooth units

The percentage of participants with insufficient mastication was significantly higher in those who underwent traditional extractions than those who did not undergo traditional extractions (Table 3). The percentage of participants with self-reported periodontal symptoms was significantly different in terms of subjective masticatory satisfaction. The number of present teeth and FTU points in those who reported good masticatory ability were 24.1 teeth and 8.5 points, respectively. These numbers were significantly bigger than those who reported poor masticatory ability.

**Table 3:** Percentage of traditional examination and self-reported periodontal symptoms and mean number of tooth and FTU points by mastication

	Number	Traditional extraction		*P* value^a^	Tooth mobility		*P* value^a^	Gum bleeding		*P* value^a^	Present teeth		*P* value^b^	FTU^c^		*P* value^b^
		N	%		N	%		N	%		M0ean	Median		Mean	Median	
*Can you masticate sufficiently now?*																
No	52	49	94.2	0.04	39	75.0	0.010	29	55.8	0.03	18.7	21	< 0.01	5.8	6	< 0.01
Yes	79	64	81.0		41	51.9		28	35.4		24.1	25		8.5	10	

^a^Fisher's exact test/chi-square test

^b^Mann–Whitney U test/Kruskal–Wallis test

^c^Functional tooth units

Table 4 demonstrates the association between subjective masticatory ability and perceived general health. The percentage of participants who had good general health among those who had good subjective masticatory ability was significantly higher than those who had poor masticatory ability. The odds ratio of good perceived general health was 2.63 [95% confidence interval (CI) 1.27–5.44] for good subjective masticatory ability. Although the odds ratio adjusted by age and sex decreased to 2.29 (95% CI 1.05–4.99), it was still significant.

**Table 4:** Percentage of those who had good perceive general health and odds ratio of good perceived general health by mastication

	Total number	Good general health			Logistic analysis			Multiple logistic analysis^b^		
		N	%	*P* value^a^	Odds ratio	(95% CI)	*P* value	Odds ratio	(95% CI)	*P* value
*Can you masticate sufficiently now?*										
No	52	18	34.6	0.012	1.00			1.00		
Yes	79	46	58.2		2.63	(1.27–5.44)	0.01	2.29	(1.05–4.99)	0.04
Total	131	64	48.9							

^a^Fisher's exact test

^b^Adjusted by age and sex

## Discussion

Oral health examination including the assessment of traditional tooth extraction was performed by visiting individual homes. The prevalence of traditional tooth extraction was 86.3%, and it increased with increasing age. Satisfactory mastication was dependent on traditional extraction, number of teeth present, FTU points, and self-reported periodontal symptoms. This is the first field research to show that satisfactory mastication is independently related to perceived general health which is known to be strongly associated with mortality risk in the elderly population in rural Kenyan community.

Home visit survey was conducted at Mbita Constituency in Homa Bay County, Kenya. Almost all participants were from the Luo ethnic group. The Kenyan Luo ethnic group practice traditional tooth extraction in the six lower anterior permanent teeth as a rite of passage into adulthood [26]. Almost 90% of the participants underwent the extraction of their six lower teeth in this study. Our study showed that traditional extraction was inversely associated with the participants’ masticatory satisfaction. Regarding the subjective masticatory ability, traditional extraction may have an adverse effect on the elderly in this area.

Satisfactory mastication was dependent on the number of teeth present, FTU points, and self-reported periodontal symptoms. The association between better masticatory satisfaction and more teeth present, specifically more FTU points in molar occlusion, has been suggested by the previous studies [27, 28]. Masticatory performance and biting force have also been reported to be associated with periodontal diseases [29–31]. These results suggest that prevention for tooth loss especially at molar segments and treatment of periodontal diseases would be required to achieve masticatory satisfaction. When the present study was conducted, there were no dental clinics within the study area. The “Kenya national oral health survey report 2015” reported that there was 1 dentist per 42,000 population in Kenya and 80% of the dentist were concentrated in large urban areas [32]. In the Kenyan elderly, periodontal disease is widespread and has been shown to be a major cause of tooth loss [20, 21]. Countermeasures to expand dental healthcare services in rural areas in order to prevent tooth loss and treat periodontal diseases will be considered important for the aging society in Kenya.

In our study, the satisfactory mastication was associated with good perceived health status independent of participants' sex and age. Perceived health status in the elderly has been reported as a simple and good predictor for mortality [19, 33–35]. Maintaining satisfactory mastication may prevent early death through their good perceived health status. As mentioned earlier, satisfactory mastication was dependent on the number of teeth present, FTU points, and self-reported periodontal symptoms. The establishment of the dental healthcare system in rural Kenya is considered to be one of the priority healthcare systems to maintain a healthy life and prevent early death in the Kenyan elderly.

This study has limitations. First, diet survey was not conducted in this study. Therefore, the validity of masticatory satisfaction based on the participants’ daily meals was not measured. Second, perceived general health status and self-reported periodontal symptoms were evaluated using a questionnaire only. Clinical health condition was not assessed because health checkups including blood test were not conducted. Also, periodontal status was not assessed with periodontal probing. However, the validity of self-reported “gum bleeding” and “tooth mobility” compared with clinical standards were confirmed by the systematic reviews [36]. Third, oral health study comprising 29 dropouts could not be conducted in advance. The difference regarding age and sex between the participants and dropouts was not observed statistically. Therefore, sampling bias occurring in our study was considered to be small. Finally, the same dentist examined whether healthy teeth or decayed teeth based on WHO standards from 2014 to 2016, but an intra-examiner reliability test for quality control was not conducted between 3 years.

## Conclusions

Oral health examination was performed by visiting individual homes in a rural community in Kenya. Satisfactory mastication was dependent on traditional extraction, number of teeth present, FTU points, and periodontal symptoms. Furthermore, satisfactory mastication was associated with perceived general health status independent of sex and age.

## Data Availability

The datasets generated and analyzed during the current study are not publicly available due to ethical approval limitations involving anonymity but are available from the corresponding author on reasonable request.
